# Recent Advances in Extracellular Vesicles as Drug Delivery Systems and Their Potential in Precision Medicine

**DOI:** 10.3390/pharmaceutics12111006

**Published:** 2020-10-22

**Authors:** Bart de Jong, Eric Raul Barros, Joost G. J. Hoenderop, Juan Pablo Rigalli

**Affiliations:** 1 Department of Physiology, Radboud Institute for Molecular Life Sciences, Radboud University Medical Center, 6500HB Nijmegen, The Netherlands; Bart.deJong@radboudumc.nl (B.d.J.); eabarros@uc.cl (E.R.B.); joost.hoenderop@radboudumc.nl (J.G.J.H.); 2 Department of Endocrinology, School of Medicine, Pontificia Universidad Católica de Chile, Santiago 8320000, Chile

**Keywords:** exosomes, extracellular vesicles, drug delivery, nanoparticles, precision medicine

## Abstract

Extracellular vesicles (EVs) are membrane-bilayered nanoparticles released by most cell types. Recently, an enormous number of studies have been published on the potential of EVs as carriers of therapeutic agents. In contrast to systems such as liposomes, EVs exhibit less immunogenicity and higher engineering potential. Here, we review the most relevant publications addressing the potential and use of EVs as a drug delivery system (DDS). The information is divided based on the key steps for designing an EV-mediated delivery strategy. We discuss possible sources and isolation methods of EVs. We address the administration routes that have been tested in vivo and the tissue distribution observed. We describe the current knowledge on EV clearance, a significant challenge towards enhancing bioavailability. Also, EV-engineering approaches are described as alternatives to improve tissue and cell-specificity. Finally, a summary of the ongoing clinical trials is performed. Although the application of EVs in the clinical practice is still at an early stage, a high number of studies in animals support their potential as DDS. Thus, better treatment options could be designed to precisely increase target specificity and therapeutic efficacy while reducing off-target effects and toxicity according to the individual requirements of each patient.

## 1. Introduction

### 1.1. The Need for Advanced Drug Delivery Systems

The therapeutic efficacy of any pharmacological treatment depends on achieving an optimal drug concentration at the site of action. This is strongly influenced by the absorption, distribution, metabolism and excretion of therapeutic agents. Moreover, these processes do not exert a constant and predictable influence on the drug concentration at the site of action, but, on the contrary, are subject to a dynamic regulation. Exposure to other xenobiotics, changes in the diet and metabolism, and disease states may affect, for instance, the expression and activity of drug transporters and metabolizing enzymes and, ultimately, influence the concentration of a drug at its active site and thus, its therapeutic efficacy [1,2]. In this regard, selectively surpassing the physiological mechanisms responsible for limiting drug absorption, and more importantly, those mediating drug clearance, may increase drug bioavailability. Furthermore, the fine-tuning of drug delivery to particular tissues and to particular cells, constitutes a strategy not only to enhance the availability of a drug at the required site but also to reduce the chemical burden to other tissues.

**Figure 1:**
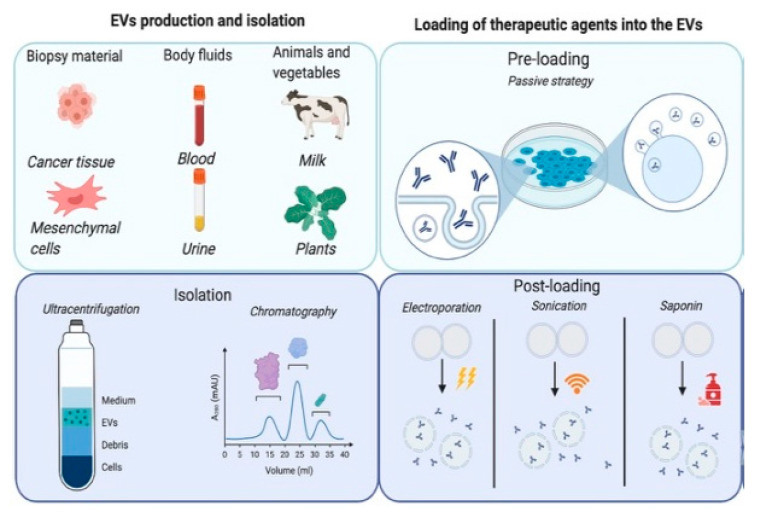

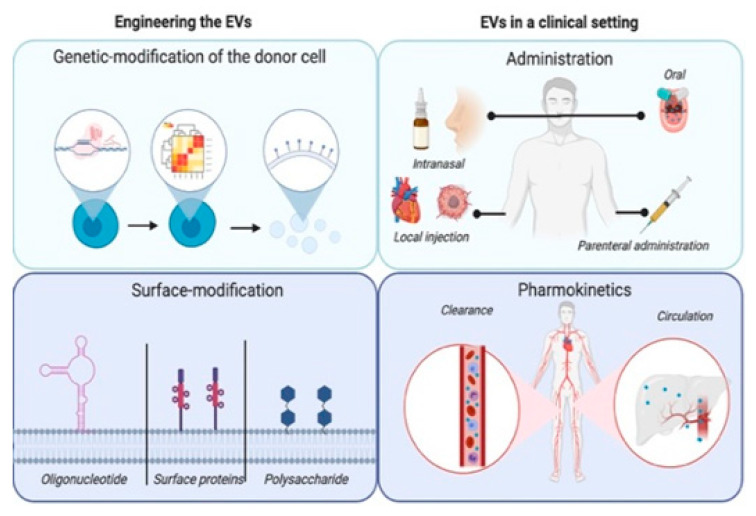
Conceptual overview of the different phases of EV-based therapy. EVs are produced and can be isolated from a wide range of cell types. EVs could be isolated from tissue biopsies, body fluids, as well as from animal and plant materials. To be used as a DDS, EVs can be isolated, for example, by ultracentrifugation or chromatography, depending on the type of EV and the source. In the second stage, the therapeutic agent will be loaded into the EVs. With pre-loading methods, the agent will be encapsulated into the EV in a natural way (i.e., using the machinery of the cell of origin). Post-loading approaches consist of physical or chemical methods, in which a disruption of the cell membrane of the already purified EV is induced. To improve the efficacy of the therapy, EVs can be modified, for instance, to increase the expression of certain receptors on the membrane, and this way its interaction with specific target cells or to avoid phagocytosis by the mononuclear phagocytic system. Finally, EVs are administered to the patients. The use of a proper route of administration may improve the pharmacokinetics of the drug by avoiding clearance or accumulation in off-target sites.

EV-based therapies could offer a significant advantage in the delivery of drugs to specific targets. This would contribute to the already on-going shift from the old paradigm of one treatment for all the patients with the same disease to a new paradigm where each patient receives a tailored therapy based on individualised parameters. This potential is reflected in the immense expansion of knowledge within this field in the past years, with high impact papers being continuously released and showing the diverse possibilities offered by EVs in different animal models. In this article, we review the latest (i.e., published in the last 5 years) and most sound studies on this novel application of EVs as a drug delivery system (DDS). Reviewed articles comprise publications describing the use of EVs as drug carriers and studies where EVs represented the therapeutic agent, as important information on the biodistribution and tissue specificity can also be gained from the latter studies. Information is presented based on the logic work-flow followed towards designing an EV-based therapy. First, the techniques for isolation and purification of EVs will be presented. Afterwards, different strategies to load EVs with therapeutic agents will be discussed. Following, we will describe possible administration routes for EV-based therapy and how tissue specificity and bioavailability can be enhanced. These different phases of an EV-based therapy are summarized in Figure 1.

### 1.2. Extracellular Vesicles

EVs comprise a heterogeneous group of nanoparticles surrounded by bilayered phospholipidic membranes, which are released by eukaryotic and prokaryotic cells into the extracellular microenvironment [3]. In eukaryotic cells, EVs can be classified based on their mode of biogenesis and release into exosomes, microvesicles and apoptotic bodies [4]. Since several isolation and characterization techniques do not allow for a clear distinction between different types of EVs, and according to the position paper of the International Society for Extracellular Vesicles [5], the term extracellular vesicle (EV) will be preferred throughout this article, even in those cases where the original literature may refer to the vesicles as exosomes.

EVs carry proteins, nucleic acids, lipids and small molecules, whereby their specific cargo depends on the microenvironment and the pathophysiological state of the cell [6]. The transfer of EVs between cells may affect cell physiology but also play a role in the mechanisms of several diseases [7,8]. Besides their role in health and disease, EVs are considered as a rich source of biomarkers for the diagnosis of several diseases. More recently, the use of EVs as a new DDS, as well as a vehicle to deliver other therapeutic agents (e.g., oncolytic viruses) to specific tissues started to be investigated.

EVs offer several advantages in comparison to other carriers. Although synthetic particles such as liposomes already entered the market more than 20 years, their use exhibits significant disadvantages, such as high toxicity [9], high clearance and immunogenicity [10]. On the contrary, besides exhibiting lower toxicity [11], EVs may undergo less phagocytosis by macrophages and can therefore remain in circulation and in the tissues for a longer time. In this regard, EVs from antigen presenting cells carry both classes of the major histocompatibility complex (MHC) molecules at relatively high expression and can therefore stimulate CD8^+^ and CD4^+^ T-cells [12,13]. However, the T-cell stimulatory effect of these free EVs is 10–20 times less efficient compared with parent antigen presenting cells [14,15]. This would explain the lower capability of EVs of stimulating naïve T-cells and, this way, their low immunogenicity. Furthermore, T-cell activation requires more than only the presence of MHC molecules but also co-stimulation and T-cell receptor cross-linking, for which not all the required components are available at the surface of the EV [12,16]. Furthermore, EVs may avoid the endosomal pathways and lysosomal degradation more effectively than synthetic carriers do [17].

## 2. Isolation and Production of EVs

### 2.1. Sources of EVs

In vitro cell culture represents a common source of EVs for therapeutic purposes. Different cell types can be targeted by EVs from this origin and different effects may be observed. For instance, preclinical data from a mice study showed that intravenous injection of EVs from bone marrow mesenchymal stem cells (MSCs) suppressed hypoxia-induced pulmonary inflammation and ameliorated pulmonary hypertension [18]. Also, EVs from dendritic cells prevented allograft rejection in a model of renal transplantation in mice [19]. In addition, macrophage-derived EVs loaded with two types of chemotherapeutic agents showed positive results against triple negative breast cancer cells in both in vitro and in vivo mouse models [20]. These findings clearly exemplify that EVs isolated from cultured cells may have an effect on different target cells in vivo. Furthermore, this also highlights the potential of this type of EVs to be loaded with therapeutic agents with the aim of their delivery to particular target cells once administered to a whole organism.

Tumor cells represent another source of EVs, particularly promising in the field of cancer therapy. So far, one of the major limitations of cancer treatment is the enhanced chemoresistance exhibited by tumor cells [21], making it imperative to develop novel therapeutic strategies. In this regard, tropism of cancer-derived EVs could be useful. However, this tropism is not uniform for all types of EVs. In fact, some types of EVs are taken up by tissues or cell types different than the ones they originate from [22]. For instance, it was demonstrated that there is a heterologous and cross-species tumor-tropism for cancer-derived EVs. The uptake of lung cancer-derived EVs by colon carcinoma cell lines and vice versa was demonstrated in in vitro and in vivo models. Also, EVs derived from human lung cancer cells were taken up by breast tumors in a mice model [23]. This suggests that there could be a general tropism of cancer EVs for other neoplastic tissues. Towards, providing a personalized treatment strategy, special effort should be directed towards targeting the treatment to a particular cell type and reducing off-target effects. In this regard, the applications of EVs on cancer treatment have been extensively reviewed by Burgio et al. [24].

Regarding EV production from cell culture, it is important not only to carefully define the best cell source, but also the media, growth conditions, sera, type of culture (e.g., 2D or 3D), metabolic preconditioning and other culture conditions [25]. In this regard, a recent study described the difference between cells grown in a soft 3D matrix in comparison with those grown in a 2D plastic surface. Noteworthy, cytospin-A, an important cytoskeleton protein was described to regulate the softness of the EVs. The physical softness of the EVs from tumor-repopulating cells obtained from a 3D culture makes them more capable to penetrate and extravasate into the tumor parenchyma in a higher concentration and deliver more efficiently chemotherapeutic drugs. Therefore, cytospin-A could be a crucial player to improve the in vivo transport of EVs isolated from 3D cultures, eventually, leading to a better treatment outcome [26].

Cancer EVs can be isolated from fluids such as plasma [27,28,29]. For prostate cancer [30] and bladder cancer [31] urine may also constitute a source of vesicles. Also, gastric juice has been reported as a source of EVs in gastric cancer patients [32]. Finally, cancer cells cultured in vitro represent another useful source of EVs [33]. With the aim of treatment with autologous EVs, a tumor biopsy may be obtained as a source for the generation of the EVs. This approach may bear the advantage of delivering a higher number of specific cancer cell-derived EVs, than when vesicles are isolated from other biofluids.

Besides cancer cell EVs, other types of EVs can also be isolated from blood. Different types of therapeutic agents have already been loaded into blood-derived EVs like miRNAs [34], curcumin [35], or dopamine [36]. Serum derived EVs have demonstrated to deliver molecules in a more efficient manner than macrophages cell-derived EVs. Especially, foetal bovine serum-derived EVs were able to enter both the macrophage- and T cell-zones within lymph nodes, also displaying an increased delivery of stimulating biomolecules [37]. Other accessible fluids are urine [38] and saliva [39]. While the use of urinary EVs as a DDS has been briefly evaluated [40], the possibilities of saliva-derived EVs in a DDS are yet not known.

Furthermore, EVs can be isolated from vegetables, fruits and milk. EVs derived from bovine milk can be used to deliver anti-cancer drugs in rats and mice without toxic effects. Furthermore, this source of EVs may allow for scaling up production in a cost-effective way [41]. Similarly, ginger roots were used as EV source for administration in mice [42]. An extensive summary of all sources of EVs that have being used, including those used for in vivo administration, can be found in Table 1 and Table 2.

### 2.2. Isolation of EVs

So far, no universal isolation protocol has been established and different applications may require different types of EVs and, therefore, different isolation techniques. Currently available methods are based on ultracentrifugation, size chromatography, immunoaffinity capture and precipitation, whereby variable purity and yield can be obtained. Different isolation methods for EVs as well as their advantages and disadvantages have been reviewed elsewhere [43]. A frequent limitation of techniques based on size and centrifugation is the frequent overlap between different types of EVs, such as microvesicles and exosomes [44,45]. Thus, the use of these isolation methods requires multiple characterization assays before it can be concluded that a specific subset of EV was isolated [5].

Aqueous two-phase systems consisting of two mixed polymers with or without a salt solution have been described as an alternative to isolate EVs. For instance, polyethylene glycol (PEG) and dextran (DEX) were dissolved into urine and centrifuged at 1000× *g*. This resulted in the separation of the two polymers resulting in a top phase containing the PEG-rich solution and a bottom DEX-rich phase containing the EVs [46]. In comparison with the earlier mentioned techniques, the purity and the efficiency are higher and no specialized equipment is required, thus suggesting the potential of this approach to be scaled for bulk production.

### 2.3. Loading of EVs with Therapeutic Agents

#### 2.3.1. Post-Loading Approach

This strategy is based on the loading of the therapeutic agent to EVs previously isolated from cells or another source of vesicles. It can be used, mainly, for synthetic molecules or small biomolecules. EV loading can take place passively or actively. The concept of passive loading is that isolated EVs are incubated with the therapeutic molecules, which will permeate into the EVs without additional stimulation. Active loading techniques are based on the application of a stimulus or adjuvant and can be divided into physically- and chemically-induced loading. Physically active methods such as electroporation, sonication, or extrusion disrupt the EV membranes to transfer the drug into the EV. Chemicals like saponin or transfection reagents can also be used to penetrate the membrane of the EV. A summary of the different post-loading methods as well as the therapeutic agents incorporated in each case is presented in Table 1.

#### 2.3.2. Pre-Loading Approach

**Figure 2:**
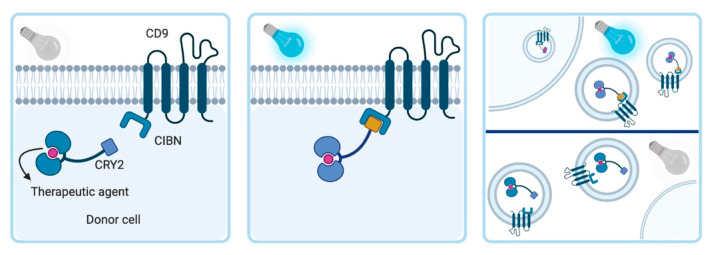
Principle of the EXPLORs technique. The binding between the CD9 complex and the CRY2 coupled to the therapeutic protein is induced in the donor cell by exposure to blue light (488 nm). Within 15 s, the cargo-protein is guided to the inner surface of the cell membrane and binds to the CIBN. The secreted EVs can be isolated, purified, and used for therapeutic purposes. Abbreviations: CIBN: truncated version of CRY-interacting basic-helix-loop-helix 1 (CIB1) protein; CRY2: Cryptochrome 2.

In the pre-loading approach, the cellular machinery is used to load a therapeutic agent into the EVs during the vesicle biogenesis. As a result, secreted EVs will carry the therapeutic agent. One pre-loading alternative is the transfection or transduction of genetic material codifying a product of interest into EV-producing cells. As a consequence, the product will be overexpressed and encapsulated in the EVs. An example of this type of loading was described by Yuan et al. [47]. In this study, the genetic sequence of the tumor necrosis factor-related apoptosis inducing ligand (TRAIL) was transduced with a lentivirus and expressed in mesenchymal stromal cells. The secreted EVs from these cells carried TRAIL and induced apoptosis with high efficiency and selectivity in three different cancer cell lines. Noteworthy, with this type of loading method, it may be difficult to control the concentration that is loaded into the EVs as they may depend on multiple factors including transfection or transduction efficiency and cell viability. Hereby, models stably expressing the product of interest may represent an advance towards obtaining a more standardized pre-loading into the EVs. For the pre-loading of EVs with a protein cargo, an innovative approach is the technique called ‘exosomes for protein loading via optically reversible protein-protein interactions’ (EXPLORs) [48]. The principle is based on two different proteins. Cryptochrome 2 (CRY2) is fused covalently to the intended cargo protein. The second component is the exosome-associated tetraspanin CD9 bearing a special variant of cryptochrome-interacting basic-helix-loop-helix 1 (CIBN). The fusion between these two structures is induced by excitation with blue light (488 nm). Following the formation of EVs, the light source is removed. Thus, the CIBN complex detaches from the CD9 and results in the release of the cargo protein of interest into the intraluminal space. This approach may be particularly useful to enrich the EVs with therapeutic proteins which are not loaded into the EVs under physiological conditions [48]. The EXPLOR method is graphically depicted in Figure 2.

The preloading strategy may also be applied to load vesicles aimed at delivering oncolytic viruses. Although this type of viruses can selectively infect and kill cancer cells, the host immune system may detect their presence and neutralize them, thus interfering with the desired effect [49]. Gould et al. [50] described the use of EVs as a virus carrier. Virus-loaded EVs induced infection in cancer cells, which also led to the infection of other autologous cancer cells. This principle was also tested in vivo in a lung cancer xenograft model and also combined with the chemotherapeutic agent paclitaxel. The combination showed a significantly higher antitumoral efficacy in comparison to the virus alone [51].

## 3. Administration and Biodistribution

Different routes have been evaluated for EV administration in experimentation animals (Table 2). Intravenous administration constitutes the most frequent route and has been reported for the administration of vesicles isolated from different sources, whereby mesenchymal stem cells (MSCs) constitute the most common one [98,99]. EVs obtained from this source have been more extensively studied for their potential in regenerative medicine (reviewed by Campanella et al., [100]). However, they may also be used for the treatment of other disorders. Intravenous administration of MSC-derived EVs resulted in the presence of EVs or in the observation of EV-related effects in a wide range of organs and tissues. Noteworthy, the delivery of therapeutic agents to the central nervous system (CNS) constitutes a challenge due to the structural properties of the blood brain barrier as well as the presence of drug extruding transporters in the brain endothelial cells, impairing the penetration of xenobiotics [101]. Hereby, intravenous administration of EVs from MSCs has been repeatedly successful towards the delivery of EVs, for instance, carrying different miRNAs, to the CNS [59,102,103,104]. Similarly, other studies described the intravenous delivery to the CNS of EVs isolated from adipose tissue stem cells [105], HEK293T cells [106], urinary stem cells [40], dendritic cells [107] and rat serum [108]. However, it is worth mentioning that most of these did not evaluate whether the same experimental approach may result in effects in other tissues. In this regard, other studies described the intravenous delivery of EVs from MSC and adipose stem cells to other tissues such as such as pulmonary vasculature [109], carotid artery [110], heart [111], lungs [112], pancreas [113], liver [114] and colon [115]. Based on this evidence, intravenous administration of EVs from MSC or adipose stem cell does not appear to provide enough specificity to target one particular tissue or organ. For this purpose, further refinements of the production strategy may be required, for example vesicle engineering (see Section 5).

Alternatively, intravenous administration of EVs isolated from sources with a closer relation to the target tissue may be a possibility towards obtaining a higher specificity. Recent reports described the delivery of urinary EVs to the kidney [116], EVs from Schwann cells to peripheral nerves [117], serum EVs from mice with acute lung injury to the lungs [118] and EVs from cardiac progenitor cells to the heart [119,120,121]. Also, intravenous delivery to the CNS has been observed for vesicles isolated from microglial cells [122,123] and mouse brain endothelial cells [124].

The intravenous route has been also reported to be useful in the delivery of cancer-derived EVs to tumoral tissue [125]. Interestingly, administration of tumor exosome-based nanoparticles loaded with doxorubicin resulted in a reduction in tumor volume and increased survival time in mice xenograft models for hepatocellular carcinoma and for breast cancer, when compared to other therapeutic alternatives. Importantly, for hepatoma xenografts, the antitumoral effect of the nanoparticles was even higher than that of intravenous free doxorubicin. In addition, the use of the same nanoparticles resulted in a reduction in lung metastasis in a mice xenograft model for melanoma. Importantly, the systemic toxicity of the nanoparticle-based strategy was lower than for the administration of free doxorubicin [126]. Conversely, it is well-acknowledged that EVs, as key players in cell-cell communication, may play a role in cancer pathogenesis, promoting initiation and progression [127]. Further research should aim at elucidating the safety of EV-based cancer therapy approaches, especially in long-term studies. Furthermore, a deeper knowledge of the role of EVs in cancer pathogenesis and the components of the cargo responsible for the tumor-stimulatory effects, may also contribute to the engineering and design of effective and safe EV-based therapeutic strategies for cancer.

Intranasal administration represents the second most frequently reported route. Delivery of MSC-derived EVs to the CNS has been described in several studies [128,129,130,131,132]. In line with these findings, Scholl et al., [133] described the intranasal delivery of glioma-derived EVs obtained in vitro to glioma tissue in rats. The advantages of this administration route may be particularly important in the treatment of neurodegenerative disorders [134]. Also, successful intranasal EV delivery to the lungs has been reported [135,136]. To date, comparative evidence of the intranasal route versus other administration routes is limited. In a recent report, Bonafede et al., [105] compared the administration of adipose stem cell-derived EVs intranasally and intravenously in a murine model of amyotrophic lateral sclerosis. Most protective effects were achieved similarly both for the intravenous and the intranasal administration. On the contrary, the intranasal administration was more effective in reducing astrocyte activation [105].

The subcutaneous administration of EVs has been described for the generation of a systemic immune response in mice administered with vesicles isolated from *Echinostoma caproni* [137]. Also, subcutaneous administration of EVs from adipose stem cells resulted in an alleviating effect in a mice model of atopic dermatitis. Here, comparative experiments were performed and similar results were observed for the intravenous and the subcutaneous pathways [138]. Systemic effects have also been reported for the intramuscular administration of EVs in chicken [139]. In addition, the intramuscular injection of EVs resulted in the delivery of vesicles to the kidneys [140]. As expected, also local effects of the vesicles at the muscular level were observed [141,142].

A comparative study from Brossa et al., [99] described, however, a reduced accumulation of MSC and liver stem cells derived EVs in subcutaneous renal tumors following intraperitoneal administration respect to the accumulation after intravenous administration. Also, Zhou et al. [143] compared both administration routes and observed an enrichment of intravenously-administered EVs in liver, spleen and lungs. Intraperitoneal administration resulted in a more disperse distribution pattern, whereby visceral adipose tissue displayed a significant accumulation of vesicles. Based on these findings, this strategy could be used in the treatment of diseases associated to visceral adipose tissue (e.g., metabolic syndrome).

Oral administration was described for EVs from bovine-milk in a mice model. Six hours post-administration, the vesicles were localized in the liver, heart, spleen, lungs and kidneys [144]. Furthermore, a comparative study between oral and intravenous administration of bovine-milk EVs described an enrichment of intravenously administered vesicles in the spleen, liver and, to a lower extent, lungs of mice. Conversely, the enrichment took place primarily in the liver, when the same EVs were administered orally [145]. These findings highlight the oral route as a possible strategy to simplify the use of EVs as DDS, without requiring specialized healthcare personnel.

Local injection of EVs may reduce the off-target delivery and increase the exposure of the target tissue and reduce the clearance of the vesicles, compared to systemic administration. For instance, intraocular and subconjunctival injection of MSC-derived EVs resulted in the delivery of the vesicles to the retina in a rabbit model of diabetes-induced retinopathy [146]. Intramyocardial injection has also been used for MSC-derived EVs, in a rat model of acute myocardial infarction [147]. Also, intrathecal injection was described for the delivery of EVs to peripheral nerves in rats [148]. Likewise, intratumoral administration resulted in a clear enrichment of EVs in the tumor, absence of EVs in liver and spleen and reduced clearance, compared with the intravenous administration [149].

In addition to the route of administration, EV size is an additional factor that could influence the biodistribution profile. For example, larger EVs accumulated in higher concentrations in bones and lymph nodes compared to smaller EVs. Nonetheless, the contribution of the molecular compositions of the EVs to this accumulation profile cannot be excluded [150].

An extensive summary of the individual studies performed, highlighting the source of EVs, their route of administration and the tissues affected under each treatment strategy can be found in Table 2.

## 4. EV Clearance

One of the key mechanisms involved in the clearance of EVs is their uptake by the mononuclear phagocytic system. Intravenously administered EVs have shown to be rapidly cleared from blood circulation, followed by hepatic, splenic and lung accumulation [209]. Interestingly, the intravenous administration of EVs carrying an siRNA against the clathrin heavy chain reduced EV uptake by the liver and the spleen and increased accumulation in the cardiac tissue [210]. In another approach, tumor derived nanovesicles were taken up by Kupffer cells and prevented the phagocytosis of doxorubicin-loaded liposomes, which instead were efficiently delivered to the lungs [211]. Similar strategies could be used to prevent the phagocytosis of EVs loaded with therapeutic agents.

Surface proteins may also be engineered to overexpress particular surface proteins and, thus, increase EV bioavailability. For instance, cluster domain (CD) 47 is an integrin-associated transmembrane protein that protects EVs from phagocytosis and thereby increase their time of circulation. In a pancreatic ductal adenocarcinoma model, EVs with the higher expression of CD47 had a longer half-life time in circulation than particles with lower CD47 [212].

## 5. Tissue Specificity

Although several studies have shown a successful delivery of therapeutic agents via EVs (Table 2), EVs could still be engineered not only to become more invisible to the host’s immune system but also to increase tissue- or cell-specificity. In fact, the accumulation of EVs at off-target sites can induce unwanted effects, thus influencing the efficacy and safety of the treatment. Coating EVs with synthetic materials or increasing the expression of certain surface proteins have been proposed as strategies to enhance the tissue specificity of the vesicles. This could ultimately lead to a more personalized treatment strategy.

PEG-coating of EVs increased the vesicle bioavailability as well as their extravasation ability, thus increasing the accumulation in tumoral tissue [213]. Kooijmans et al., [214] described the use of pegylated-targeting ligands. In particular, coating of the EVs with PEG-EGFR increased their bioavailability and enhanced the EV binding to EGFR-overexpressing tumor cells, while decreasing non-specific interactions. Similarly, coating macrophage-derived and paclitaxel loaded EVs with PEG-aminoethylanisamide, which targets the sigma receptor (usually overexpressed in lung cancer cells), was effective delivering EVs to pulmonary metastases in mice [85].

Streptavidin can be used in combination with PEG as an anchor point, and biotinylated-components (e.g., antibodies, homing peptides) targeting cells of interest can be then conjugated to the EV surface. This rather simple approach increased the uptake of EVs by cardiac fibroblasts, myoblasts and cardiomyocytes exposed to ischemic conditions, suggesting that it could be a promising strategy to increase cell-specificity not only in the heart but also in other organs [215].

Finally, the use of magnetic fields to direct EVs to a particular target has also been reported. This approach was applied to direct superparamagnetic iron oxide nanoparticle-covered EVs loaded with an experimental peptide to pancreatic islet cells to increase insulin secretion. Interestingly, the peptide loaded in the EVs showed increased plasma half-life and stronger glucose-lowering effects than the free peptide [216]. Following, the most common approaches to engineer the EV surface and thus modulate target specificity, will be discussed.

### 5.1. Homing Peptides

The display of homing peptides has been used to direct intravenously administered EVs to the CNS. Here, the cyclo(Arg-Gly-Asp-D-Tyr-Lys) peptide, which exhibits high affinity for an integrin highly expressed in ischemic brain, was added to the EV surface via click-chemistry. This strategy resulted in a significant enrichment of EVs carrying curcumin in the brain compared to non-decorated EVs [217]. A similar strategy was effective to direct EVs carrying miR-210 to ischemic brain areas [59]. Another peptide used for the same purpose is a short sequence of the rabies virus glycoprotein (RVG), which has been expressed in the EV-generating cells coupled to the exosomal protein Lamp2b. This resulted in the generation of EVs exhibiting a higher tropism for the central nervous system [218]. The same strategy was used to direct HEK293T EVs to the mouse brain [219]. A similar RVG peptide was used by Cui et al., [220], although by chemical coupling with dioleoylphosphatidylethanolamine N-hydroxysuccinimide to anchor the peptide to the surface of the vesicle. Noteworthy, another study described the presence of intravenously administered EVs displaying the same RVG peptide but in muscle and kidney tissues [140]. Therefore, although this approach appears to increase the EV affinity for brain tissues, binding to other organs cannot be ruled out.

Homing peptides have also been used to direct EVs to cardiac tissue. This was reported by conjugating a cardiac homing peptide to the surface of EVs isolated from cardiac stem cells via a dioleoylphosphatidylethanolamine N-hydroxysuccinimide (DOPE-NHS) linker [121]. These decorated EVs exhibited an increased uptake of EVs by cardiomyocytes. However, the use of cardiac stem cell-derived EVs may have also contributed to this observation. Also, the decoration of EVs with another cardiac targeting peptide coupled to the EV membrane via Lamp2b increased the delivery to cardiac tissue in a mice model [221].

Peptides have also been used to increase delivery to tumor cells, such as triple negative breast cancer cells. In this case, a peptide targeting the mesenchymal-epithelial transition factor (c-Met) was conjugated to the surface of doxorubicin-loaded EVs via a 1,2-distearoyl-sn-glycero-3-phospho-ethanolamine-N-[methoxy(polyethylene glycol)-2000] link. After intravenous administration, the peptide-coated EVs exhibited a significant enrichment in the tumor tissue and displayed higher antitumor efficacy as compared to other nanoparticles or even free-doxorubicin [222]. It must be noted, however, that coated-EVs were also detected in other tissues (e.g., spleen, liver, lungs). The same principle was applied in another study where the EGFR-specific peptide GE11 was used to direct intravenously administered EVs to a mammary tumor in nude mice. Here, the coating was performed through overexpression in the EV-generating cells. A significant enrichment in the tumoral tissue was observed for GE11-EVs compared to control vesicles. Furthermore, the authors demonstrated the absence of significant damage to other organs, thus pointing towards the safety of this strategy [166]. Similarly, Tian et al., [168] demonstrated a higher antitumor efficacy of doxorubicin-loaded EVs in mice bearing MDA-MB-231 cell tumors when the EVs were coated with the iRGD peptide coupled to Lamp2b. The effect can be attributed to the specific interaction iRGD with the αv integrin in the tumoral cells. No cardiac damage was detected. This observation could be beneficial in terms of developing therapeutic strategies to minimize the cardiotoxicity usually associated to the treatment with doxorubicin.

**Figure 3:**
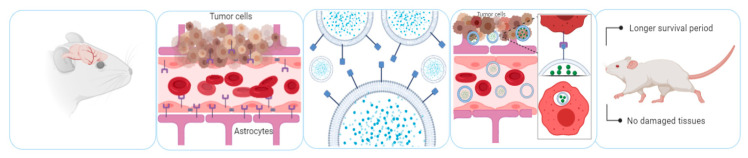
Engineering of the EVs to increase target specificity. Blood-brain-barrier, glia and tumor cells expressed the low-density lipoprotein (LDL) receptor. EVs loaded with methotrexate were conjugated with a surface peptide targeting the LDL receptor. In a mice model, administration of these modified EVs resulted in longer survival period and no damage of other tissues, as frequently observed with conventional chemotherapy strategies [178].

This strategy was also used as therapeutic alternative for glioblastoma multiforme. Both the blood-brain-barrier and glioma cells overexpress the low-density lipoprotein receptor on their surface. EVs were loaded with the anti-cancer agent methotrexate and conjugated with a peptide that targets the overexpressed lipoprotein receptor. Glioblastoma-bearing mice that were treated with these modified EVs showed a longer median survival period compared to the control groups including methotrexate alone and EVs loaded with methotrexate but without coating [178]. Noteworthy, no lesions or damage to other tissues were observed. This strategy, as an example of increased delivery to the CNS, is summarized is Figure 3.

### 5.2. Protein Ligands

Surface proteins play a key role in the interaction between EVs and target cells. For instance, intravenous injection of EVs from cardiac progenitor stem cells overexpressing the C-X-C chemokine receptor type 4 (CXCR4) improved cardiac function in a rat model of ischemia/reperfusion injury when compared with control EVs without CXCR4 overexpression [120]. Additionally, the interaction of the integrin LFA1 on the surface of the EVs with ICAM1 on the cell surface appears to play a key role in the vesicle uptake by brain endothelial cells [177]. Also, the interaction between fibronectin on the vesicle surface and heparan sulfate on the cell surface mediates the interaction between EVs and myeloma cells [223].

Another related strategy is the addition of antibodies to the EV surface. For instance, the display of an anti-HER2 antibody on the surface of the vesicles increased the delivery of EVs to HER2^+^ breast cancer cells when administered intraperitoneally to mice [224]. A similar antibody-based approach was used in a mouse model of colorectal cancer where doxorubicin was delivered to tumor cells via EVs. In this case antibodies against the A33 antigen were used. Noteworthy, the antibodies were not added directly to the surface of the EVs but to superparamagnetic iron nanoparticles, thus allowing a magnetically-directed delivery to the tumor region while increasing also the specificity for tumor cells. Again, no cardiac damage was observed, as compared to the strategies without A33 antibody addition or to free doxorubicin [52]. Similarly, intravenous administration of EVs displaying an HIV-1 specific monoclonal antibody and loaded with curcumin resulted in a higher growth inhibition of solid tumors expressing the HIV-1 envelope protein (Env) in mice. Since most HIV-1 infected cells display viral proteins on their surface, this strategy bears an enormous potential for precision HIV antiviral therapy [164].

### 5.3. Nucleic Acids

DNA and RNA molecules displayed on the cell surface may also be used to increase target specificity. For instance, a DNA aptamer displayed on EVs from bone marrow stromal cells was used to enhance the delivery of EVs to the bone in mice which received EVs intravenously [163]. Synthetic RNA nanoparticles can be added to the surface of the EVs as a platform to incorporate further tissue-specific ligands. This was described for folic acid. When the EVs were loaded with a therapeutic siRNA against survivin, coupling to folic acid resulted in a higher inhibition of the tumor growth in a mice xenograft model for human epidermoid carcinoma, as compared with vesicles without folic acid on their surface. The increased specificity could be explained based on the interaction between folic acid on the EV surface and the folate receptor in the tumor cells [42].

### 5.4. EV-Surface Glycosylation

The glycosylation of surface proteins plays an important role in EV-cell interaction. It was described that the treatment of EVs with neuraminidase, which removes the terminal sialic acid residues of surface proteins, affects the distribution of EVs after administration to mice. A higher enrichment in the lungs was observed when neuraminidase-treated EVs were administered intravenously respect to the untreated EVs. Similarly, when neuraminidase-treated EVs were administered into the hock, the distribution among the lymph nodes displayed a different pattern respect to untreated EVs [225].

A different strategy is modifying the surface of the EVs with cationized pullulan, a polysaccharide that consists of maltotriose-units. Tamura et al., [226] used cationized pullulan to enhance the electrostatic interaction and uptake of EVs by liver cells. After administering pullulan-modified EVs to a mouse model of liver injury, there was an improved anti-inflammatory and tissue-regenerative effect in comparison with the original vesicles. The effects could be explained in terms of the interaction between pullulan and asialoglycoprotein [226]. Similarly, decoration of doxorubicin-loaded EVs with hyaluronan-PEG resulted in a differential uptake of the vesicles to breast cancer and lung adenocarcinoma cells in vitro. Notably, the vesicle-based strategy led to a higher inhibition of cell growth respect to the treatment with free doxorubicin. These findings could be explained at the molecular level due to the interaction between hyaluronan and CD44, overexpressed in the surface of cancer cells [227]. Similarly, serum-derived exosomes incorporating mannose-conjugated PEG-1,2-distearoyl-sn-glycero-3-phosphoethanolamine in their lipid bilayer showed high accumulation in mannose-receptor expressing dendritic cells and lymph nodes [228].

## 6. Clinical Grade Production

Translation of the previously described findings, mostly obtained in experimentation animals, to the clinical practice requires standardized good manufacturing practices (GMPs). Several challenges arise at all the steps of the production and have been recently reviewed by Wiest et al., [229]. The selection of the cellular sources and the characterization of the vesicles represent one of the first challenges. Here, adhering to the position paper of the ISEV on the minimal information for studies of extracellular vesicles 2018 (MISEV 2018) should be the starting point for defining production strategies [5]. Furthermore, clinical grade production requires adherence to strict quality and safety regulations. Hereby, the International Council for Harmonisation of Technical Requirements for Pharmaceuticals for Human Use (ICH) develops guidelines on quality, safety and efficacy. Regarding regulatory requirements for EV based therapeutics, considering the complexity and challenges associated with EVs, a case by case regulatory approach seems more plausible for the evaluation of EVs as DDS rather than an all-purpose approach. Currently there are no specific regulatory guidelines for EVs, although they have to be filed and regulated as drugs and biological products according to the Food and Drug Administration (FDA), depending on the origin, isolation procedure, route of administration, active substance, or mechanism of action, and are subjected to premarket review and approval. Further discussion on the regulatory challenges to perform clinical trials using EVs can be found in a recent position paper from the ISEV [230] and have been reviewed elsewhere [231].

So far, only a few studies described the production and characterization of clinical-grade EVs following GMPs. Previously, Kamerkar et al., [212] highlighted the potential of EVs loaded with an siRNA against the mutated form of the RAS kinase. In a more recent study of the same group, the generation of clinical-grade EVs following GMPs from the selection of the donor to the isolation and loading was presented [232]. Here, the authors not only succeeded in the upscaling of the loading electroporation protocol to higher sample volumes but also demonstrated the efficacy of the preparation in a mouse-model. Also, importantly, the authors confirmed the stability of the EVs (i.e., number and size distribution) after freezing at −80 °C for 45 days or 6 months. Storage at room temperature or 4 °C for 2 or more days resulted in a decrease in the therapeutic efficacy assessed in vitro. The successful design and validation of this strategy led to the register of a clinical trial (NCT03608631, see Section 7). The replacement of animal materials in the production process is an important requirement for clinical grade EVs. In this regard, FCS can be replaced by human platelet lysates depleted of EVs [233].

Production of large volumes of high purity EVs is an important challenge. Here, a combination of tangential flow filtration (TFF) and size exclusion chromatography (SEC) exhibited significant advantages respect to other isolation methods [234]. EVs carrying the heterodimeric interleukin-15 were isolated from HEK293 cells cultured in hollow fiber bioreactors. The combination of both isolation techniques resulted in a lower contamination with proteins without decreasing the yield. Similarly, a comparative study between TFF and ultracentrifugation highlighted the higher batch-to-batch reproducibility in the size distribution of EVs isolated by TFF. Moreover, removal of protein contaminants was not only 40 times higher using TFF but also exhibited less variability than ultracentrifugation. Noteworthy, EV suspensions obtained by TFF also tested negative for mycoplasma, bacteria and endotoxin [235]. The successful use of TFF for the production of EVs from bone marrow MSCs and ASCs was also reported [236,237]. Altogether, TFF appears as a useful tool in terms of obtaining high purity clinical grade EV suspensions with high reproducibility between batches.

The previous studies established production pipelines for clinical grade EVs and provide a proof-of-concept on the feasibility of different processes. Other studies described extensive quality controls (QCs) that would be required for industrial production. For instance, the study of Andriolo et al., describing GMP-compliant EV production from cardiac progenitor cells, described exhaustive QC tests. First, the stability of the cell source was controlled by establishing a post-production cell bank (i.e., cells at the limit of the time, in which they are suitable for production) only for QC purposes. Comparisons between this cell bank and the master cell bank using in the regular production constitute an important QC. Furthermore, the authors perform an extensive QC on two batches of EVs in terms of sterility, presence of endotoxin, particle concentration and size, expression of exosome markers and total protein. Also, functional checks (e.g., proangiogenic activity) displaying comparable results with both lots of EVs were presented [238]. Likewise, another study analyzed the batch-to-batch variability of EVs obtained from ASCs through TFF. High reproducibility was registered in terms of size and surface markers and purity [239].

Hitherto, a wide variety of vesicles have been used to target different tissues and organs in experimentation animals. However, only a few of these vesicle types have been validated for clinical grade production. One critical aspect that needs special attention is, besides those presented above, the effect of interindividual variability of the cell donors on the batch-to-batch variability of the produced EVs. In addition, while GMP-grade culture media and supplements and TFF-mediated isolation led to the production and validation of different EV suspensions, it is yet unclear whether this constitutes a cost-effective alternative. Finally, more comparative studies addressing the impact of the different strategies for the preparation and storage of clinical grade EV formulations on their safety and potency are also required.

## 7. Clinical Trials

Different high-quality studies performed in animal models support the potential of EVs as a DDS from the point of view of the safety and efficacy at the preclinical level. At the clinical level, the application of EVs is still at a very early stage. So far, 18 clinical trials involving therapeutic use of EVs have been registered at the NIH website [240] (Table 3). Noteworthy, two studies evaluate the use of EVs as DDS. In particular, the study NCT01294072 plans to evaluate the use of plant-derived EVs for curcumin delivery to normal and colon cancer, while the NCT03608631 study plans to evaluate the delivery of a therapeutic siRNA to pancreas cancer tissue. Although the rest of the studies do not foresee the use of vesicles as drug carriers, they could provide information concerning the tolerability of EV administration, suitability of different administration routes (e.g., topical, intravenous, oral, inhalation) and use of different sources of EVs (plasma, plants, MSCs, adipose tissue stem cells). Although no results of the current studies are yet available, this information may later be used towards optimizing the use of EVs for delivering therapeutic agents in clinical practice.

## 8. Future Perspectives and Conclusions

In the last ten years, the understanding of the role of EVs in cell communication and their potential as a source of biomarkers have been acknowledged and further explored. More recently, there has been an increase in the body of evidence addressing EV secretion, biodistribution and specificity. These findings point to a significant potential of EVs to be used as a DDS. This novel delivery strategy shows high promise, although many questions remain unanswered regarding EVs, along with technical and experimental challenges that hinder the translation from the laboratory to clinical trials.

One still remaining challenge is assuring the safety of EV-based treatments. Although several studies in animal models indicate a high tolerability and ongoing clinical trials will evaluate this in humans, further research should be performed to rule out potential deleterious effects of the carrier EVs in cell-cell communication. Hereby, the use of plant EVs may have an advantage by carrying a natural cargo less related to human EVs. While the delivery potential would still be present, the chance of triggering physiological or pathophysiological responses (e.g., immune response) may be lower.

Another challenge regarding EV research, is target specificity. So far, several engineering possibilities have been evaluated. Unfortunately, while many studies nicely describe an increased targeting of certain organs and tissues, off-target delivery is not always evaluated. Furthermore, future research regarding surface molecules mediating the interaction between EVs and target cells will contribute towards increasing the delivery specificity for EVs. Hereby, the characterization of the surface proteome of the EVs and the surface glycan profile of target cells could significantly improve EV target specificity.

In summary, the design of therapeutic strategies using EVs as a DDS will require the optimization of all the steps of involved in the EV pipeline. Safe and efficient sources of EVs, along with isolation and loading techniques that are cost-efficient and allow to scale production, in combination with the validation of proper administration routes and EVs that are specific for a particular target, remain the cornerstone in EV research. In this way, EVs could be proposed as a new alternative in precision medicine, aimed at increasing therapeutic efficacy and decreasing side effects, resulting in improved quality of life for the patients.

## Figures and Tables

**Table 1:** Strategies for the loading of EVs with therapeutic agents. Detailed are different studies describing the loading of EVs with therapeutic agents. The specific loading method, the source of EVs as well as the therapeutic agent or experimental cargo are mentioned. Subsequently, the method used to evaluate the loading efficiency, if known, is indicated. Finally, the model where the generated EVs were applied is indicated. Abbreviations: DNA-CH: cholesterol-coupled DNA; dsDNA: double stranded DNA; EXPLORs: exosomes for protein loading via optically reversible protein-protein interactions; hESC: human embrionary stem cell; HPLC: High-performance liquid chromatography; HUVEC: human umbilical vein endothelial cell; miRNA: micro RNA; LC-MS: Liquid chromatography–mass spectrometry; MRSA: Methicillin-resistant *Staphylococcus aureus*; MSC: mesenchymal stem cell; siRNA: small interfering RNA; ssDNA: single-stranded DNA; TAMEL: Targeted and modular EV loading; t-PA: tissue-plasminogen activator; UPLC: Ultra-Performance Liquid Chromatography.

Loading Method	EV Source	Therapeutic Agent or Experimental Cargo	Loading Efficiency	Model	Ref.
Passive incubation	Cell culture (LIM1215 cells)	Doxorubicin	Spectrofluorometry	Cell culture, mice tumor xenograft	[52]
	Cell culture (HEI-OC1 cells)	Aspirin, arachidonic acid, eicosapentaenoic acid, docosohexaenoic acid, linoleic acid, lipoxin, resolvin D1			[53]
	Cell culture (HEI-OC1 cells)	Dexamethasone			[54]
	Cell culture (HEK293T cells)	siRNA and rhodamine	Spectrofluorometry		[55]
	Cell culture (LNCaP and PC-3 cells)	Paclitaxel	UPLC	Cell culture	[56]
	Cell culture (LL/2, MC-38, A549 cells), liver tissue	Oncolytic adenovirus		Cell culture and mice with colon adenocarcinoma xenograft	[23]
	Cell culture (MDA-MB-231, MSCs, hESCs, HUVECs)	Different porphyrins		Cell culture	[57]
	Cell culture (MSCs)	Doxorubicin	Spectrophotometry	Cell culture	[58]
	Cell culture (MSC)	miR-210		Mice	[59]
	Cell culture (MSC)	Nanoparticles loaded with curcumin	Spectrophotometry		[60]
	Cell culture (Neuro2A, dendritic cells)	siRNA	Spectrofluorometry	Cell culture	[61]
	Cell culture (Raw 264.7 cells)	Linezolid	HPLC	Mice with MRSA infection	[62]
	Cell culture (Raw 264.7 cells)	Paclitaxel, doxorubicin	HPLC	Cell culture, different xenograft mouse models	[20]
	Cell culture (Raw 264.7 cells)	Paclitaxel, doxorubicin	HPLC	MDCK-MDR1 cells; mice	[63]
	Cell culture (Raw 264.7 cells)	Catalase	Catalase enzymatic activity	Cell culture, mice	[64]
	Cell culture (THP-1)	Doxorubicin, t-PA, photosensitizer molecules	Spectrofluorometry	Cell culture	[65]
	Cell culture (U87 cells)	Paclitaxel	HPLC	Cell culture	[66]
	Platelets	Doxorubicin	Spectrofluorometry	Cell culture	[67]
	Bovine milk	Withaferin, anthocyanidins, curcumin, paclitaxel, docetaxel	Spectrophotometry, UPLC	Rats, mice with different xenograft variants	[41]
	Bovine milk and cell culture	Curcumin		Cell culture	[68]
	Blood	Dopamine	LC-MS	Mice	[36]
Electroporation	Cell culture (Dendritic cells)	Let-7-miRNA and siRNA	qPCR	Mice with breast tumor xenograft	[69]
	Cell culture (HEI-OC1 cells)	Aspirin, arachidonic acid, eicosapentaenoic acid, docosohexaenoic acid, linoleic acid, lipoxin, resolvin D1			[53]
	Cell culture (HEK293T cells)	Doxorubicin	Spectrofluorometry	Cell culture, mice with breast tumor xenograft	[70]
	Cell culture (HEK293T cells)	siRNA, rhodamine	Spectrofluorometry		[55]
	Cell culture (HEK293T cells)	siRNA	Spectrofluorometry	Cell culture	[71]
	Cell culture (HEK293T cells)	siRNA		Cell culture	[72]
	Cell culture (HEK293T cells)	Doxorubicin	Spectrofluorometry	Cell culture	[73]
	Cell culture (HEK293T cells, HUVECs)	dsDNA	Spectrophotometry	Cell culture	[74]
	Cell culture (MDA-MB-231, MSCs, hESCs, HUVECs)	Different porphyrins		Cell culture	[57]
	Cell culture (MDA-MB-231 cells)	Superparamagnetic iron nanoparticles and olaparib		Cell culture, mice with breast tumor xenograft	[75]
	Cell culture (MSCs)	Doxorubicin	Spectrofluorometry	Mice with multiple xenograft variants	[76]
	Cell culture (Normal intestinal fetal human cells)	miRNA-128-3p	qPCR	Cell culture, mice with colon tumor xenograft	[77]
	Cell culture (Raw 264.7 cells)	Superparamagnetic iron nanoparticles loaded with curcumin	Spectrophotometry	Mice bearing glioma cells	[78]
	Cell culture (Raw 264.7 cells)	Paclitaxel, doxorubicin	HPLC	MDCK-MDR1 cells; mice	[63]
	Cell culture (3T3 and A549 cells)	siRNA		Cell culture	[79]
	Plasma and cell culture	Curcumin, siRNA, DNA-CH, miR-145 mimics		Mice with lung tumor xenograft	[35]
	Plasma	miR-31-5p, miR-451a		Cell culture,	[34]
	Serum	Tyrosinase-related-protein-2	Spectrofluorometry	Cell culture	[80]
Sonication	Cell culture (HEI-OC1 cells)	Dexamethasone			[54]
	Cell culture (HEK293T, MCF7 cells)	siRNA, miRNA, ssDNA		Cell culture	[81]
	Cell culture (IC21 cells)	Tripeptidyl peptidase-1	Enzymatic activity	Cell culture, mice	[82]
	Cell culture (J774A.1 cells)	Doxorubicin		Cell culture	[83]
	Cell culture (Human fetal lung fibroblast 1)	Erastin	HPLC	Cell culture	[84]
	Cell culture (Raw 264.7 cells)	Catalase	Catalase enzymatic activity	Cell culture, mice	[64]
	Cell culture (Raw 264.7 cells)	Paclitaxel	HPLC	Cell culture, mice	[85]
	Cell culture (Raw 264.7 cells)	Paclitaxel, doxorubicin	HPLC	MDCK-MDR1 cells; mice	[63]
	Cell culture (Raw 264.7 cells)	Paclitaxel, doxorubicin	HPLC	Cell culture, mice with breast cancer xenograft	[20]
	Cell culture (U937 cells)	Dexamethasone	HPLC	Cell culture, mice	[86]
	Cell culture (U87 cells)	Paclitaxel	HPLC	Cell culture	[66]
Lipofection	Bovine milk	siRNA		Zebrafish and mice	[87]
Transfection	Cell culture (A172 cells)	siRNA	RT-PCR	Mice	[88]
	Cell culture (HEK293T cells)	Anti-miR-214		Cell culture, mice	[89]
	Human peripheral blood cells	miR-21		Cell culture, mice	[90]
Saponin permeabilization	Cell culture (IC21 cells)	Tripeptidyl-peptidase-1	Enzymatic activity	Cell culture, mice	[82]
	Cell culture (MDA-MB-231, MSCs, hESCs, HUVECs)	Different porphyrins		Cell culture	[57]
	Cell culture (Raw 264.7 cells)	Catalase	Catalase enzymatic activity	Cell culture, mice	[64]
	Serum	Tyrosinase-related-protein-2	Spectrofluorometry	Cell culture	[80]
pH-gradient	Neutrophils	Piceatannol	HPLC	Cell culture, mice	[91]
Extrusion	Cell culture (MDA-MB-231 cells, MSCs, hESCs, HUVECs)	Different porphyrins		Cell culture	[57]
	Cell culture (Raw 264.7 cells)	Catalase	Catalase enzymatic activity	Cell culture, mice	[64]
Freeze-thaw cycles	Cell culture (Raw 264.7 cells)	Catalase	Catalase enzymatic activity	Cell culture, mice	[64]
Hypotonic dialysis and extrusion	Cell culture (MDA-MB-231 cells, MSCs, hESCs, HUVECs)	Different porphyrins		Cell culture	[57]
Transfection	Cell culture (HEK293T cells)	miR-21 sponge	RT-qPCR	Cell culture, rats	[92]
	Cell culture (HEK293T cells)	miR-199a-3p	RT-qPCR	Mice	[11]
	Cell culture (HEK293T cells)	miR-199a-3p	RT-qPCR	Cell culture	[93]
	Cell culture (HEK293T cells)	Cre recombinase	RT-qPCR	Cell culture	[94]
	Cell culture (IC21 cells)	Tripeptidyl-peptidase-1	Enzymatic activity	Cell culture, mice	[82]
	Cell culture (Normal intestinal fetal human cells)	miRNA-128-3p	qPCR	Cell culture, mice with colon tumor xenograft	[77]
	Cell culture (MSCs)	Anti-miR-222/223		Mice with breast tumor xenografts	[95]
	Cell culture (4T1, SKBR3, HepG2 cells)	Anti-miR-21	Fluorescence microscopy	Cell culture	[96]
TAMEL	Cell culture (HEK293T cells)	RNA		Cell culture	[97]
ExPLORs	Cell culture (HEK293T cells)	Recombinant luciferase	Luciferase activity	Cell culture, mice	[48]

**Table 2:** Administration routes for EVs in vivo. Detailed are different studies using in vivo models where EVs were administered. Described are the administration routes, the experimental model, the source of EVs, the therapeutic cargo (when known), and the target tissue of the EVs or the EV-related effects. While in some cases a therapeutic cargo was known, in other cases the EV itself was the therapeutic agent and no particular component of the cargo was specified as responsible for the effect observed. Abbreviations: AdSC: adipose tissue-derived stem cells; CNS: central nervous system; miR: micro RNA; MSC: mesenchymal stem cells.

Administration Route	Experimental Model	Source of EVs	Therapeutic Cargo	Target Tissues	Reference
Intravenous	Mice	Plasma		Liver	[151]
		Serum	miR-124	CNS	[152]
		Serum		Lung	[118]
		Serum		Systemic effects	[153]
		Mice serum, supernatant of cultured myotubes	miR-21	Kidney	[154]
		Blood	Dopamine	CNS	[36]
		Urine	Klotho	Kidney	[116]
		Milk		Liver, spleen, heart, lungs	[145]
		MSC	Paclitaxel	Subcutaneous tumors and distant metastases	[155]
		MSC		Heart	[111]
		MSC		Liver	[156]
		MSC	miR-210	Brain	[59]
		MSC	miR-let7	Atherosclerotic plaque	[157]
		MSC	miR-125b	Heart	[158]
		MSC		Bone marrow	[159]
		MSC		CNS	[160]
		MSC, liver stem cells		Subcutaneous tumor	[99]
		AdSC	miR-199ª	Orthotopic tumor	[161]
		AdSC		CNS	[105]
		AdSC	miR-17	Liver	[114]
		AdSC		Skin	[138,162]
		Bone marrow stromal cells		Liver, lungs, bone	[163]
		HEK293T cells	Anti-miR-214	Subcutaneous tumor	[89]
		HEK293T cells	Curcumin, miR-143a	Tumor cells	[164]
		HEK293T cells	miR-199a-3p	Subcutaneous tumor	[165]
		HEK293 cells		Mammary tumor	[166]
		Dendritic cells		Spleen	[167]
		Dendritic cells	siRNA	Brain	[107]
		Immature dendritic cells	Doxorubicin	Mammary tumor	[168]
		Mouse brain endothelial cells	miR-126	CNS	[124]
		Endothelial colony forming cells	miR-486-5p	Kidney	[169]
		Gastric epithelial cells		Aorta	[170]
		Neural primary stem cells		CNS	[171]
		BMD2a cells		Lungs, liver, spleen, brain	[172]
		Liver	miR-130a-3p	Systemic effects	[173]
		Schwann cells		Peripheral nerves	[117]
		Astrocytes		CNS	[174]
		Microglial cells	miR-124-3p	CNS	[122]
		Breast cancer cells	miR-126	Lung cancer cells	[125]
		Tumor-cell exocytosed-exosome biomimetic porous silicon nanoparticle	Doxorubicin	Tumor cells	[126]
		Gastric cancer cells		Blood myeloid-derived suppressor cells	[175]
		Pancreas carcinoma cells		Liver, spleen, lungs	[176]
		Macrophages	Brain-derived neurotrophic factor	CNS	[177]
		L929 cells	Methotrexate	Glioblastoma tissue	[178]
		Ginger roots	siRNA	Subcutaneous tumor	[42]
	Rats	Serum		CNS	[108]
		MSC	miR-544	CNS	[102]
		MSC	CC chemokine receptor type 2	CNS	[179]
		MSC		CNS	[180]
		MSC		CNS	[181]
		MSC	miR-17-92 cluster	CNS	[104]
		MSC		Pulmonary vasculature	[109]
		MSC		Colon	[115]
		MSC		Vein graft	[182]
		MSC		Heart	[183]
		MSC		Lungs	[112]
		MSC	miR-29b	Spinal cord	[184]
		MSC		Pancreas	[113]
		MSC		Carotid artery	[110]
		MSC		Bone	[185]
		AdSC		Penile tissue	[186]
		AdSC	miR-126	CNS	[187]
		AdSC		Brain, spleen	[188]
		AdSC		Systemic effects	[189]
		AdSC		CNS	[190]
		Human urinary stem cells	miR-26a	CNS	[40]
		Human fetal amniotic fluid stem cells		Heart	[191]
		Cardiac progenitor cells	miR-146a	Heart	[119]
		Cardiac progenitor cells		Cardiomyocytes	[120]
		Cardiac stem cells		Heart	[121]
		Human iPSCs		Bone	[192]
		Urinary stem cells		Kidney	[193]
		Aortic adventitial fibroblasts	miR-155-5p	Aorta and mesenteric artery	[194]
		HEK293T cells	miR-21 antisense	CNS	[106]
		HEK293T cells	siRNA	CNS	[195]
		Human renal tubular cells		Kidney	[196]
		Renal cells		Kidney	[197]
	Patients with COVID-19	MSC		Systemic effects	[198]
	Monkeys	MSC		CNS	[199]
Intravenous (in utero)	Ovine fetuses	MSC		Brain	[200]
Intranasal	Mice	MSC		CNS	[201,202,203]
		AdSC		CNS	[105]
		ESC	Curcumin	CNS	[204]
		Astrocytes	siRNA	Microglia	[88]
		Amnion epithelial cells		Lungs	[205]
		Macrophages		Lungs	[135,206]
		EL4, 3T3L1, 4T1, CT26 and A20 cells	Curcumin, JSI-124	CNS	[132]
		Bronchoalveolar lavage fluid		Lungs, systemic effects	[136,207]
	Rats	MSC		CNS	[128,130,131,208]
		MSC	siRNA	CNS	[129]
		C6 Glioma cells		Glioma	[133]
		Human teeth stem cells		CNS	[134]
Intraperitoneal	Mice	Liver stem cells, MSC		Subcutaneous tumor	[99]
Intramyocardial	Rats	MSC		Heart	[147]
Subcutaneous	Mice	AdSC		Skin	[138]
		*Echinostoma caproni*		Systemic immune response	[137]
Intramuscular	Mice	Primary mouse satellite cells	miR-29	Kidney, muscle	[140]
		Cardiac stem cells		Muscle	[141]
		Human iPSCs		Muscle	[142]
	Chicken	Serum		Systemic immune response	[139]
Intrathecal	Rats	MSC		Peripheral nerves	[148]
Oral	Mice	Bovine milk		Liver, spleen, heart, lungs, kidney	[144]
Intraocular	Rabbit	MSC		Retina	[146]
Subconjunctival	Rabbit	MSC		Retina	[146]

**Table 3:** Clinical trials registered at clinicaltrials.gov using EVs for therapeutic purposes. Studies using EVs as a drug delivery system are highlighted in bold. Abbreviations: AdSCs: adipose tissue stem cells; MSCs: mesenchymal stem cells; SARS-CoV-2: several acute respiratory syndrome coronavirus 2.

EVs	Disease or Condition	Route of Administration	NCT Number
Plant EVs loaded with curcumin	Colon cancer	Oral	NCT01294072
MSCs EVs loaded with KRAS G12D siRNA	Pancreas cancer	Intravenous	NCT03608631
MSCs	Healthy individuals	Inhalation	NCT04313647
MSCs	SARS-CoV-2	Inhalation	NCT04276987
MSCs	Acute ischemic stroke	Stereotaxic injection	NCT03384433
MSCs	Macular holes	Intravitreous injection	NCT03437759
MSCs	Dystrophic epidermolysis bullosa	Topical	NCT04173650
MSCs	Depression, anxiety, neurodegenerative disorders	Intravenous	NCT04202770
MSCs	Bronchopulmonary dysplasia	Intravenous	NCT03857841
MSCs	Type 1 diabetes mellitus	Intravenous	NCT02138331
MSCs	SARS-CoV-2	Inhalation	NCT04491240
MSCs	Alzheimer disease	Intranasal	NCT04388982
MSCs	SARS-CoV-2	Intravenous	NCT04493242
AdSCs	Periodontitis	Local injection	NCT04270006
Plasma	Cutaneous wounds	Topical	NCT02565264
Not specified	Craniofacial neuralgia	Epineural injection, intravenous	NCT04202783
Not specified	Acute myocardial infarction	Intracoronary	NCT04327635
Plant	Oral mucositis associated with chemoradiotherapy	Topical	NCT01668849
